# Effects of low-intensity bodyweight training with slow movement on motor function in frail elderly patients: a prospective observational study

**DOI:** 10.1186/s12199-018-0693-4

**Published:** 2018-01-31

**Authors:** Kanae Kanda, Takeshi Yoda, Hiromi Suzuki, Yugo Okabe, Yutaka Mori, Kunihisa Yamasaki, Hiroko Kitano, Aya Kanda, Tomohiro Hirao

**Affiliations:** 1 Department of Public Health, Kagawa University Faculty of Medicine, 1750-1 Ikenobe, Miki-cho Kita-gun, Kagawa 761-0793 Japan; 2 Sin Cire Co., Ltd, 14-29 Ogi-machi Daito, Osaka, 574-0033 Japan

**Keywords:** Bodyweight training, frail elderly, LST, Motor function

## Abstract

**Background:**

Slow-motion training, an exercise marked by extremely slow movements, yields a training effect like that of a highly intense training, even when the applied load is small. This study evaluated the effects of low-intensity bodyweight training with slow movement on motor function in frail, elderly patients.

**Methods:**

Ninety-seven elderly men and women aged 65 years or older, whose level of nursing care was classified as either support required (1 and 2) or long-term care required (care level 1 and 2), volunteered to participate. Two facilities were used. Participants in the first facility used low-intensity bodyweight training with slow movement (the LST group, *n* = 65), and participants in another facility used machine training (the control group, *n* = 31). Exercises were conducted for 3 months, once or twice a week, depending on the required level of nursing care. Changes in motor function were examined.

**Results:**

Post-exercise measurements showed significant improvements from the pre-exercise levels after 3 months, based on the results of the Timed Up and Go test (*p* = 0.0263) and chair-stand test (*p* = 0.0016) in the low-intensity exercise with slow movement and tonic force generation (LST) group. Although the ability to stand on one leg with eyes open tended to improve, no significant change was found (*p* = 0.0964).

**Conclusions:**

We confirmed that carrying out LST bodyweight training for 3 months led to improvements in ambulatory function and lower-limb muscle strength. In this way, it is possible that LST training performed by holding a bar or by staying seated on a chair contributes to improved motor function in elderly patients within a short time.

**Trial registration:**

UMIN000030853. Registered 17 January 2018. (retrospectively registered).

## Background

Resistance training, which consists of applying resistance to muscles, is effective for the prevention of muscle weakness and muscle atrophy associated with aging [1, 2]. However, in conventional resistance training, no apparent muscle hypertrophy can be achieved unless high load intensity equivalent to approximately 80% of the maximum muscle strength is used [3]. Notably, high-load resistance training is associated with small, but real, risks of muscle or joint injuries and cardiovascular events [4]; therefore, muscle training methods for elderly subjects need to be safe to perform under a relatively low load.

Recently, low-intensity exercise with slow movement and tonic force generation (LST) [5], involving movements while maintaining muscle tension that allows for the obtainment of a substantial muscle hypertrophy effect using a relatively light load, has been developed, and evidence of its effects continue to be accumulated. LST training is a collective term, which refers to a practice of muscle training in which the load is increased and decreased extremely slowly. LST training has a positive effect on muscle hypertrophy and demonstrates an enhancing effect on muscle strength, even with load strength equivalent to 50% or less of the maximum muscle strength [5–9]. However, studies on this method of training conducted on elderly subjects are in their accumulation stage and have predominantly been conducted on healthy elderly subjects, with the use of muscle training machines. Accordingly, a slow-motion bodyweight training program that can be easily performed by frail elderly subjects at home is needed.

Therefore, the purpose of this study was to evaluate the effects of LST bodyweight training on motor function in frail elderly patients.

## Methods

### Participants

**Fig. 1:**
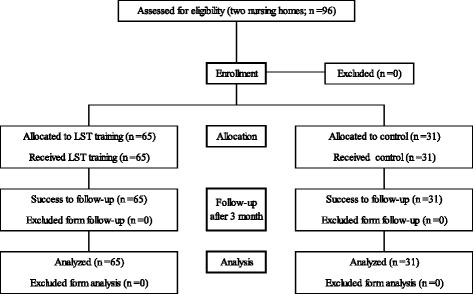
Flowchart of the participants

The study included elderly men and women aged 65 years or older who were using nursing care facilities in Osaka from March to August 2016 as well as those who required a level of nursing care classified as either support required (1 and 2) or long-term care required (care level 1 and 2). Two facilities were involved. The participants in the first facility started receiving an exercise rehabilitation therapy using LST bodyweight training (the LST group), and the participants in the other facility started receiving an exercise rehabilitation therapy using machine training (the control group). The study flowchart is shown in Fig. 1. Exclusion criteria for the participants were as follows: (1) those who had difficulty participating in the exercise program due to apparent cognitive symptoms and (2) those who had physical limitations due to some other reason. Exercise programs and exercise machines were provided by Sin Cire Co., Ltd.

### LST bodyweight training group

**Fig. 2:**
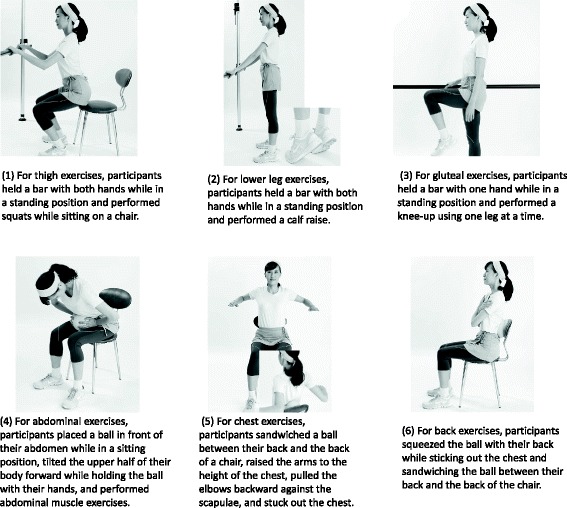
LST bodyweight training program (six components). 1—For thigh exercises, participants held a bar with both hands while in a standing position and performed squats while sitting on a chair. 2—For lower leg exercises, participants held a bar with both hands while in a standing position and performed a calf raise. 3—For gluteal exercises, participants held a bar with one hand while in a standing position and performed a knee-up using one leg at a time. 4—For abdominal exercises, participants placed a ball in front of their abdomen while in a sitting position, tilted the upper half of their body forward while holding the ball with their hands, and performed abdominal muscle exercises. 5—For chest exercises, participants sandwiched a ball between their back and the back of a chair, raised the arms to the height of the chest, pulled the elbows backward against the scapulae, and stuck out the chest. 6—For back exercises, participants squeezed the ball with their back while sticking out the chest and sandwiching the ball between their back and the back of the chair

The LST bodyweight training program was developed on the basis of the LST [5], and for 3 months, exercises were conducted at a frequency of once or twice per week, depending on the participating individual’s required level of nursing care. Regarding the amount of physical exercise, two sets of six different types of exercise (thighs, lower legs, buttocks, abdomen, chest, and back) were performed at a pace of eight times per minute (the extremities were elevated for 3 s and then lowered for 3 s), with the sets separated by 1-min breaks. Specifically, the training contents were as follows (Fig. 2): (1) for thigh exercises, participants held a bar with both hands while in a standing position and performed squats while sitting on a chair; (2) for lower leg exercises, participants held a bar with both hands while in a standing position and performed a calf raise; (3) for gluteal exercises, participants held a bar with one hand while in a standing position and performed a knee-up by using one leg each time; and (4) for abdominal exercises, participants placed a ball in front of their abdomen while in a sitting position, tilted the upper half of their body forward while holding the ball with their hands, and performed abdominal muscle exercises. Additionally, (5) for chest exercises, participants sandwiched a ball between his/her back and the back of a chair, raised the arms to the height of the chest, pulled the elbows backwards against the scapulae, and stuck out the chest, and (6) for back exercises, participants squeezed the ball with his/her back while sticking out the chest and sandwiching the ball between the back and the back of the chair. All exercises were conducted by combining slow movements and 3-s intervals of rhythmic breathing. Each participant was instructed to consciously maintain muscle activity as long as possible. All participants were instructed to follow the same exercise program under the guidance of a specialized instructor.

Participants were excluded (1) if they discontinued the ongoing program, (2) when it became difficult for the participant to continue the exercise program on a regular basis, (3) when the attending physician determined that the participant had to stop using day services because of the effects of diseases, or (4) other people whom the physician in charge of the research program determined that the patient was inappropriate for inclusion.

### Machine training group (control)

The control group was subjected to low-intensity machine training using a normal speed. The machine training consists of a program combining body area-specific muscle strength training exercises using a hydraulic weight-training machine [10–12]. Regarding the amount of physical exercise, two sets of seven different types of exercise (thighs, lower legs, buttocks, chest, back, shoulders, and abdomen) were performed at a pace of 15 times per minute (the extremities were elevated for 1 to 2 s and then lowered for 1 to 2 s), and the sets were separated by 1-min breaks. Details of the muscle training contents are as follows: (1) for the exercise of the thighs (femoral region), flexion and extension movements of the knee joint were performed by using a Leg Extension/Curl^®^ machine (GH-104; ALPS Electric Co., Ltd., Tokyo, Japan); (2) to exercise the lower legs, up-and-down movements of the lower extremities were performed using a Stepster^®^ (TB-699; Takada-Bed Co., Ltd., Sennan, Japan); (3) to exercise the buttocks (gluteal region), abduction and adduction movements of the hip joint were performed using an Abduction/Adduction^®^ (GH-102; ALPS Electric Co., Ltd., Tokyo, Japan); (4) to exercise the chest (thoracic region), a chest press was performed using a home gym machine; (5) to exercise the back (dorsal region), a pulldown was performed by using a multi-homed trainer; (6) to exercise the shoulders, arm raising and lowering movements were performed using a multi-homed trainer; and (7) to exercise the arms, arm curls were performed using a multi-homed trainer. A single training device (YMHT-250; Yamato Human Co. Ltd., Tokyo, Japan) was used for the latter four exercises.

### Measurements

The primary outcome was the difference in mean values obtained from the Timed Up and Go test (TUG, for evaluating the ability to perform compound motions). To determine TUG results [13], we measured the time required to rise from a chair and stand up, walk to a landmark located 3 m ahead, and to return to sit in the chair again. For each training exercise, measurements were conducted twice, and the best value was recorded.

The secondary outcomes were the differences in mean values obtained from the chair-stand test (lower-limb muscle strength) and the one-leg standing test with eyes open (balance ability). The chair-stand test was performed using the CS-30 test [14], and the number of times the participant sat down and stood up within a 30-s period was measured once. For the one-leg standing test with eyes open [13], we measured the time during which the participant was able to detach their foot from the floor and maintain it in the same position. A duration of 60 s was considered as the maximum, and each measurement was performed twice.

Other outcomes included participants’ age at study initiation, sex, level of nursing care required, body weight, and blood pressure at rest. The body weight of each participant was measured using a Tanita body composition meter (BC-705N; Tanita Corp., Tokyo, Japan). The blood pressure of each participant at rest (systolic pressure and diastolic pressure) was measured in a sitting position using a Terumo electronic blood pressure monitor P2000 (ES-P2000; Terumo Corp., Tokyo, Japan) after the participant had rested for at least 5 min.

### Sample size calculation

To observe a significance level alpha = 0.05, power = 0.8, standard deviation = 5.0, and effect size = 3.0, which reflects the average difference in the TUG, which is the primary outcome, each group was estimated to require 45 subjects [14–19]. The sample size was calculated using JMP Pro 12.0 (SAS Institute Inc., Cary, NC, USA).

### Statistical analysis

The results are expressed as the mean value ± standard deviation, minimum value, and maximum value. Comparisons between values measured before the exercises and those measured 3 months later were performed using the paired *t* test and Wilcoxon test. The comparison between the two groups in terms of the amount of variation was performed using an unpaired *t* test. A significance level for hazard ratios of less than 5% was considered significant in both cases. Statistical analysis was performed using JMP Pro 12.0.

### Ethics statement

Informed consent was obtained from the participants. In addition, this study was approved by the Ethics Committee of Kagawa University Faculty of Medicine and Graduate School of Medicine (approval number: Heisei28-128).

## Results

The baseline characteristics of the study participants are shown in Table 1. There were 65 participants in the LST group (mean age 80.6 ± 6.1 years) and 31 in the control group (mean age 80.4 ± 5.7 years). With regard to the current medical history, information on major diseases for which nursing care certification was required was collected by multiple responses. Among such diseases, joint diseases were the most frequent, followed by hypertensive diseases. All participants completed the 3-month exercise training program. Before the exercises, the values of each index showed no significant differences between the two groups. The CS-30 test was only significantly different between the two groups at baseline. In both groups, the average number of weekly exercise sessions undertaken by the study subjects was 1.8 times per week. No adverse events resulting from participation in the exercise training programs were reported.

**Table 1:** Baseline characteristics of the study participants

Baseline variables	LST group (*n* = 65)					Control group (*n* = 31)					*p* value
	Mean	±	SD	Minimum	Maximum	Mean	±	SD	Minimum	Maximum	
Female sex (%)	50 (76.9)					25 (80.6)					0.6838
Age (years)	80.6	±	6.1	66	93	80.4	±	5.7	70	91	0.8809
Body weight (kg)	54.7	±	10.3	30.4	84.3	57.3	±	11.3	36.4	80.1	0.2799
Systolic blood pressure (mmHg)	131.2	±	18.1	95	185	129.9	±	21.	95	181	0.7540
Diastolic blood pressure (mmHg)	67.6	±	10.7	41	92	68.6	±	10.7	43	89	0.6710
Timed Up and Go test (sec)	12.2	±	5.7	6.4	32.0	14.7	±	6.4	6.5	31.8	0.0567
One leg balance with an open eye (sec)	11.4	±	13.0	0.37	60.0	16.5	±	21.6	1.02	60.0	0.1534
CS-30 test (counts/30 s)	23.1	±	7.8	2	43	17.4	±	7.3	6	32	*0.0011*
Smoking status (%)	1 (1.5)					2 (6.4)					0.1997
Current medical history (multiple answers)											
Cerebrovascular diseases	16					9					
Heart diseases	13					7					
Malignant neoplasm	9					6					
Respiratory diseases	5					2					
Joint diseases	61					22					
Dementia	5					2					
Parkinson’s disease	2					0					
Diabetes	7					3					
Visual/hearing disorder	17					3					
Fracture/fall	15					8					
Spinal cord injury	1					0					
Hypertensive diseases	22					12					
Others	26					14					

Values are presented as means ± standard deviation, minimum value, and maximum value

All *p* values compared characteristics of the LST and control groups using unpaired *t* tests or Chi-squared tests

*LST* low-intensity exercise with slow movement and tonic force generation, *SD* standard deviation

Participant motor function prior to the LST training program was compared with the findings obtained 3 months after training, and the results showed significant improvements in the TUG (mean, 12.2 ± 5.7 s prior to the exercise training program and 11.6 ± 5.6 s at 3 months; *p* = 0.0263), as well as in the chair-stand test (23.1 ± 7.8 times prior to the exercise training program and 25.1 ± 7.7 times at 3 months; *p* = 0.0016). Although the ability to stand on one leg with eyes open tended to improve, no significant change was found (pre-exercise mean, 11.4 ± 13.1 s; mean, 12.8 ± 14.1 s at 3 months; *p* = 0.0964). The comparison of blood pressure at rest with the values found prior to the exercise training program showed that both the systolic and diastolic pressures had decreased significantly after 3 months of exercise. There was no significant change in body weight (Table 2).

**Table 2:** Comparisons between values measured before exercises and those measured 3 months later

	Baseline			3 months			*p* value
LST group (*n* = 65)							
Body weight (kg)	54.7	±	10.4	54.6	±	10.6	0.2840
Systolic blood pressure (mmHg)	131.2	±	18.2	122.7	±	17.4	*0.0005*
Diastolic blood pressure (mmHg)	67.6	±	10.7	63.9	±	10.7	*0.0063*
Timed Up and Go test (sec)	12.2	±	5.7	11.6	±	5.6	*0.0263*
One leg balance with an open eye (sec)	11.4	±	13.1	12.8	±	14.1	0.0964
CS-30 test (counts/30 s)	23.1	±	7.8	25.1	±	7.7	*0.0016*
Control group (*n* = 31)							
Body weight (kg)	57.3	±	11.3	57.8	±	11.4	*0.0266*
Systolic blood pressure (mmHg)	129.9	±	21.0	130.0	±	21.6	0.9687
Diastolic blood pressure (mmHg)	68.6	±	10.7	69.8	±	12.4	0.4565
Timed Up and Go test (sec)	14.7	±	6.4	13.7	±	5.7	0.1071
One leg balance with an open eye (sec)	16.5	±	21.6	15.0	±	21.0	0.6035
CS-30 test (counts/30 s)	17.4	±	7.3	18.3	±	7.6	0.2044

Data are presented as the mean ± standard deviation and mean change

A paired *t* test and Wilcoxon test were used to compare values measured before the exercises and those measured 3 months later

*p* < 0.05. *p* values less than 5% were considered significant in both cases

*LST* low-intensity exercise with slow movement and tonic force generation

Meanwhile, in the control group, body weight was the only item that showed a significant change in a comparison between pre-exercise values and findings after 3 months of exercise training.

**Fig. 3:**
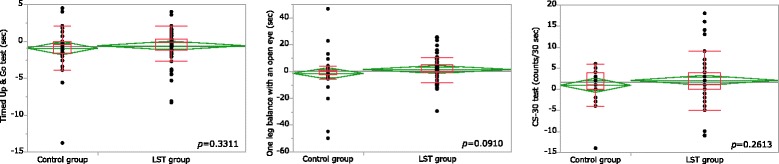
Change in motor function score following LST training. An unpaired *t* test was used to compare values between the two groups in terms of the amount of variation. Data are presented as mean (95% confidence interval). *p* < 0.05. *p* values less than 5% were considered significant in both cases. LST, low-intensity exercise with slow movement and tonic force generation

Based on the pre-exercise values and findings after 3 months of exercise, the amounts of changes showed no significant differences between the two groups (Fig. 3). There were more significant improvements in body weight, systolic blood pressure, and diastolic blood pressure in the LST group than in the control group (Table 3).

**Table 3:** Comparison between the two groups in terms of the amount of variation

	Baseline—month 3				
	LST group (*n* = 65)		Control group (*n* = 31)		*p* value
Body weight (kg)	− 0.30	(− 0.64 to 0.03)	0.49	(− 0.01 to 0.98)	*0.0105*
Systolic blood pressure (mmHg)	− 8.52	(− 12.76 to − 4.29)	0.09	(− 6.04 to 6.23)	*0.0239*
Diastolic blood pressure (mmHg)	− 3.74	(− 6.24 to − 1.23)	1.22	(− 2.40 to 4.85)	*0.0278*
Timed Up and Go test (sec)	− 0.60	(− 1.22 to 0.01)	− 0.94	(− 1.83 to − 0.04)	0.3311
One leg balance with an open eye (sec)	1.42	(− 1.51 to 4.34)	− 1.54	(− 5.77 to 2.69)	0.0910
CS-30 test (counts/30 s)	2.06	(0.91 to 3.22)	0.90	(− 0.77 to 2.58)	0.2613

An unpaired *t* test was used to compare values between the two groups in terms of the amount of variation

Data are presented as a mean (95% confidence interval)

*p* < 0.05. *p* values less than 5% were considered significant in both cases

*LST* low-intensity exercise with slow movement and tonic force generation

## Discussion

After 3 months of LST training, lower-limb muscle strength improved as did the ability to perform compound motions involved in walking. Watanabe et al. previously reported that LST training using exercise machines with a load of 30% one-repetition maximum (1RM) led to hypertrophy of the quadriceps femoris muscles in healthy elderly subjects [6]. In our study, elderly subjects were subjected to training methods using chairs and bars. Our findings showed that such types of LST bodyweight training also had an improvement effect on motor function and that the effect was similar to that of LST training completed using exercise machines. Meanwhile, although balance function tended to improve, no significant change was found with respect to this change. In a previous study that subjected elderly subjects to 12 weeks of balance training, Shimada et al. reported that an extension of the one-leg standing position-maintaining time was found in a group exposed to static balance training and that a shortening of the TUG and stair climbing and descending time was found in the group exposed to dynamic balance training; this showed a specificity of training effects [19]. The physical exercise program used in our study was a dynamic program, and this is probably the reason why no significant change was found in static balance functions, such as those measured by the one-leg stand test. In addition, to improve the balance ability of elderly women aged 75 years or older, Ml-Khoury et al. previously proposed an exercise program that could be incorporated into daily life [20]. Exercises associated with activities of daily living, including backward and forward movements of the center of gravity, might need to be added to the program in order to improve the balance function of elderly subjects [21].

In addition, in the control group, no significant change was found in any of the items after 3 months of training exercise. Although the mechanism for this is unclear, it may be difficult to obtain an improved outcome if strength training with low intensity is performed in frail elderly people at a normal iterative speed. Further, a comparison of the amounts of changes after 3 months of LST training and control, respectively, showed no significant difference in motor function between the two groups. In a comparison between an intensive 50% 1RM LST training method and a 50% 1RM conventional training method, Tanimoto et al. previously stated that significant muscle hypertrophy and an enhancing effect on muscle strength were found to be associated with LST training [22]. In our study, variations were found in the results, particularly in those pertaining to the control group, thereby suggesting that the absence of significant differences may have been due to extensive individual differences in training effect.

There were several limitations to this study. First, this was a short-term study, and so, a long-term study is needed to confirm the sustained effects of LST training. Second, the measurement items were limited to motor functions, and the study did not include any evaluation of cognitive functions or the risk of fall. Third, the underlying mechanism behind LST training’s effect is still unknown.

## Conclusions

The improvement in the lower-limb muscle strength, ambulatory function, and blood pressure at rest was confirmed as short-term effects of LST bodyweight training in frail, elderly subjects. Our findings suggest that LST bodyweight training is safe to be performed by frail, elderly subjects and that it could potentially have a short-term effect on improving motor function.

## Abbreviations

1RM: One-repetition maximum
LST: Low-intensity exercise with slow movement and tonic force generation
TUG: Timed Up and Go test
